# Stüve-Wiedemann syndrome: LIFR and associated cytokines in clinical course and etiology

**DOI:** 10.1186/1750-1172-9-34

**Published:** 2014-03-12

**Authors:** Dawn Mikelonis, Cheryl L Jorcyk, Ken Tawara, Julia Thom Oxford

**Affiliations:** 1 Boise State University, Department of Biological Sciences, Biomolecular Research Center, 1910 University Drive, Boise State University, Boise ID 83725, USA

**Keywords:** Stüve-Wiedemann syndrome, Leukemia inhibitory factor receptor, LIF, LIFR

## Abstract

Stüve-Wiedemann syndrome (STWS; OMIM #610559) is a rare bent-bone dysplasia that includes radiologic bone anomalies, respiratory distress, feeding difficulties, and hyperthermic episodes. STWS usually results in infant mortality, yet some STWS patients survive into and, in some cases, beyond adolescence. STWS is caused by a mutation in the leukemia inhibitory factor receptor (*LIFR*) gene, which is inherited in an autosomally recessive pattern. Most *LIFR* mutations resulting in STWS are null mutations which cause instability of the mRNA and prevent the formation of LIFR, impairing the signaling pathway. LIFR signaling usually follows the JAK/STAT3 pathway, and is initiated by several interleukin-6-type cytokines. STWS is managed on a symptomatic basis since there is no treatment currently available.

## Introduction

### History

Stüve-Wiedemann syndrome (STWS) was first described in 1971, presenting in two sisters with congenital bowing of the tibia and femur, abnormally positioned feet, and camptodactyly. Both patients experienced respiratory distress and consequently died within a few days of birth. One of the patients also suffered from deglutination difficulty and hyperthermia. Stüve and Wiedemann suspected that the syndrome had autosomal recessive inheritance since the patients were sisters [1,2]. Although the prognosis of STWS remains poor, recent reports show that some STWS patients survive at least into early adolescence [3,4], and beyond.

### Classification

Surviving STWS patients were found to be phenotypically identical to Schwartz-Jampel syndrome type 2 (SJS2) survivors. Therefore, SJS2 and STWS are now considered to be the same disorder, and the title “Schwartz-Jampel Syndrome type 2” has been dropped [5,6]. Both SJS2 and STWS have been linked to a mutation of the leukemia inhibitory factor receptor (*LIFR*) gene on chromosome 5p13.1 [7].

STWS includes autonomic nerve dysfunction [3], and the related ciliary neurotrophic factor receptor (*CNTFR*) gene has also been linked to autonomic nervous system dysfunction [8]. Therefore, STWS is also included in the family of CNTFR pathway-related disorders [9]. This classification exemplifies the clinical overlaps between STWS, Crisponi syndrome, which includes facial weakness, feeding difficulties, camptodactyly and hyperthermia in infancy followed by scoliosis, and cold-induced sweating in adolescence, and cold-induced sweating syndrome (CISS) [9,10]. However, the skeletal features of STWS allow it to also be classified within the group of bent-bone dysplasias [11].

STWS is found across multiple ethnic groups in multiple regions of the world including North America, Europe, and the Middle East [7,12,13]. However, STWS appears to be more common in the United Arab Emirates [14], as well as in Oman and Yemen [12,15].

## Clinical manifestations

**Figure 1:**
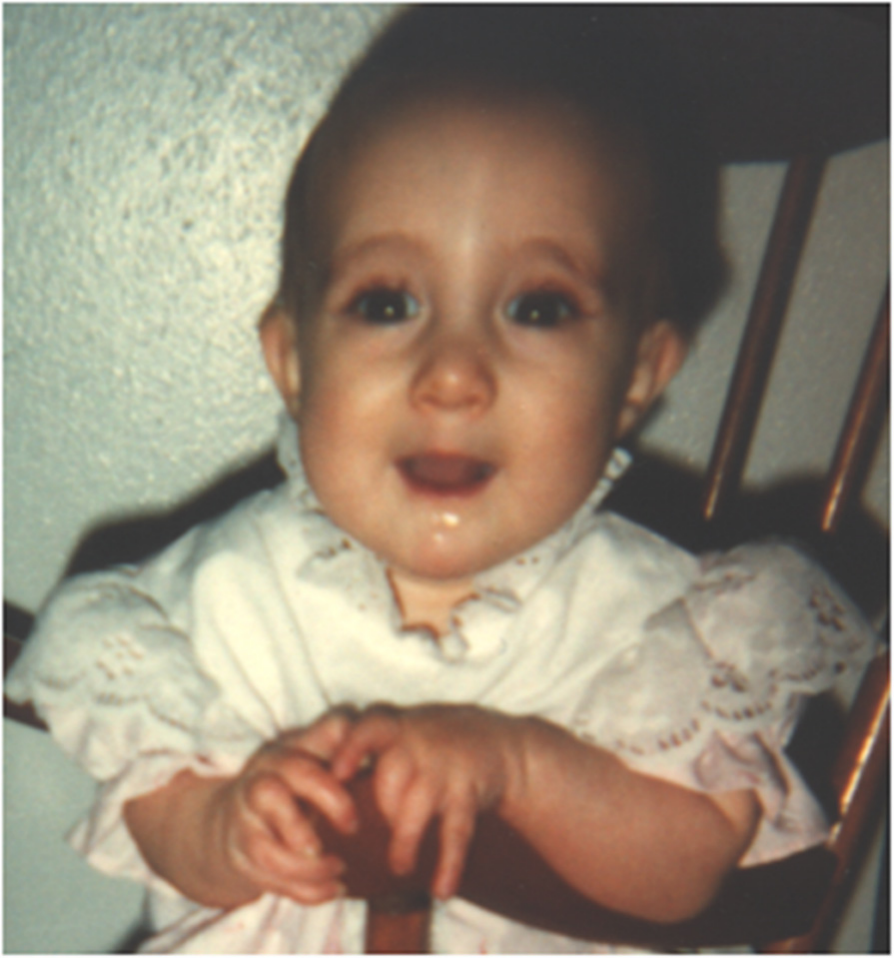
**Image of STWS patient at 10 months of age.** Facial features and contracture of fingers are evident. Patient carries two mutations in the LIFR gene; 1) a duplication of 22 nucleotides within exon 4, causing a frameshift predicted to result in a premature stop codon following five unique amino acids, and 2) a deletion of nine nucleotides within exon 12, resulting in the deletion of three amino acids at the protein level. Both mutations occur within the region of the gene encoding the extracellular domain. The mutation resulting in the premature stop codon is anticipated to result in mRNA instability, and it is therefore likely to result in a null mutation. These mutations are unique, having not been previously described. Photograph printed with permission from patient, now age 23.

STWS is a rare autosomal recessive bent-bone dysplasia (OMIM #601559). It is characterized by bowing of the long bones with cortical thickening and rarefaction, wide and blurred margins of the metaphyses, contracture and limited mobility of elbows and knees, osteopenia, flared iliac wings, hypoplasia of the lower ilia, and an abnormal trabecular shape [1,7,13,16]. Additional features include camptodactyly, contracture of fingers, and symptoms of dysautonomia (Table 1). Figure 1 shows an individual at ten months of age, who was later diagnosed with STWS. Most individuals suffering from STWS do not survive beyond the first few months of life due to respiratory distress, difficulties with feeding and swallowing, or hyperthermic episodes [1,7,10,12,13,17-19]. Patients that do survive show an improvement in prognosis as a normal breathing rhythm is established and the ability to swallow is gained, yet difficulties with swallowing can still occur later in childhood [13].

**Table 1:** Diagnostic characteristics of STWS

**Organ system**	**Neonatal**	**Childhood**	**Treatment**	**Reference**
**Embryonic**	Oligohydramnios			[20]
	Intrauterine growth restriction; low birth weight, length and head circumference	Growth retardation		[1,2,20]
**Skeletal**	Micromelia, bowing of the long bones with cortical thickening, wide metaphyses, and abnormal trabeculae	Progressive bowing of the long bones with continued radiologic abnormalities		[1,2,7,12,13,19,55]
	Camptodactyly	Camptodactyly		[1,2]
	Scoliosis	Scoliosis	Corrective surgery	[7,12,13]
	Osteopenia or osteoporosis	Osteopenia or osteoporosis	Bisphosphonates, calcium, vitamin D, human growth hormone, surgery and physical therapy	[11,13,18]
	Facial anomalies	Facial anomalies		[18]
		Spontaneous fractures Prominent joints with restricted mobility	Corrective surgery	[7,12,13,119]
**Muscular**	Hypotonia, Contractures			[19]
**Pulmonary**	Respiratory distress	Respiratory distress improves		[1,2]
	Pulmonary hypoplasia			[20]
**Cardiovascular**	Pulmonary hypertension			[79-81]
**Gastrointestinal**	Dysphagia	Dysphagia improves	Intubation, nasogastric tube feeding, gastrostomy	[12,13]
**Nervous system**	Hyperthermic episodes	Temperature instability		[1,2]
	Excessive sweating	Excessive and paradoxical sweating		[9]
	Absent corneal and patellar reflexes	Absent corneal and patellar reflexes		[7,12,13]
	Hypolacrimation	Hypolacrimation	artificial tear drops and ointments, lacrimal punctum dilation	[17,120,121]
		Delayed motor development Reduced pain sensation Smooth tongue		[7,12,13]
**Ocular**	Corneal opacities	Corneal opacities	Keratectomy	[17,120]
**Endocrine system**	Ectopic thyroid Hypothyroidism			[18]

Open cells in the table indicate no available treatment. Some features generally improve over time (i.e., respiratory distress and dysphagia), while other features become progressively worse (i.e., bowing of the long bones).

With age, there is progressive bowing of the long bones with tubulation of diaphyses, rarefaction and striation of metaphyses, and destruction of the femoral heads [12]. Severe spinal deformities arise, along with streaky osteoporosis and spontaneous fractures. The joints are prominent and show restricted mobility. Temperature instability remains, and there is excessive, and sometimes paradoxical sweating. Other signs of dysautonomia persist, such as a smooth tongue, absent corneal and patellar reflexes, and reduced pain sensation. Physical growth and motor development is delayed, but intelligence is normal [7,12,13].

In some cases, it is possible to predict the presence of STWS before birth via ultrasound. Prenatal symptoms can sometimes be seen in the late second- or third trimester. The prenatal symptoms of STWS include oligohydramnios, intrauterine growth restriction despite normal Doppler findings about the umbilical artery, camptodactyly, bowing of the lower bones affecting the tibia more than the femur, and micromelia [20].

## Genetic etiology

### Genetic mutations responsible for STWS

Dagoneau et al. determined that the leukemia inhibitory factor receptor (LIFR) gene on chromosome 5p13.1 is responsible for STWS [7]. Their findings were reinforced by the observation that *LIFR*^*-/-*^ mice have reduced fetal bone volume, an increased number of osteoclasts, reduced numbers of astrocytes in the spinal cord and brain, and perinatal death [21]. The mouse phenotype is considered to be similar to that of STWS [7]. Furthermore, most patients with STWS have a mutation within the *LIFR* gene [20]. Many STWS patients in the United Arab Emirates have an identical frameshift mutation in the LIFR gene that results in a premature stop codon [20].

The mutated *LIFR* gene is inherited in an autosomal recessive pattern. The mutations reported have been either missense or nonsense mutations, with the majority being nonsense mutations within the exons encoding the extracellular domain [7]. Fourteen distinct null mutations were observed in 19 families, most of which resulted in premature stop codons which altered the stability of the mRNA, resulting in an absence of the LIFR protein and impairment of the LIFR signaling pathway [7]. The *LIFR* gene has 19 exons and normally encodes a transmembrane protein which is 1,097 amino acids long [7,22,23].

### Genetic variability

Interestingly, not all patients who are diagnosed with STWS have an identified *LIFR* mutation [13,19]. The gene(s) responsible for the STWS phenotype in these exceptional cases have not been identified. This variability suggests that modifier genes may play a role in STWS, as well [13]. Other genes within the candidate region (chromosome 5p13.1 from locus D5S194 to D5S1457) mapped by Dagoneau et al. included *FLJ39155* (EGFLAM or Pikachurin, a proteoglycan), disabled-2 (*DAB2*), complement 9 (*C9*), Fyn-binding protein (*FYB*), and oncostatin M receptor (*OSMR*) [7]. While these genes may play a role as disease modifiers or may be potential candidates for those cases of STWS that are not linked to a mutation in LIFR, to date, limited information exists to support the likelihood of the involvement of these genes in skeletal, respiratory or neurological symptoms associated with the syndrome. For example, a neurologic function has been described for the *FLJ39155* locus. *FLJ39155* gene product has been localized to the synaptic cleft of the photoreceptor ribbon synapse and gene disruption experiments demonstrated the necessity of this protein for proper synaptic signal transmission and visual function [24]. Knockout of this gene selectively disrupts synaptogenesis between photoreceptor and bipolar cells. Additionally, micro-duplications of the short arm of chromosome 5 that include the 5p13.1 region have been linked to neurologic symptoms [25]. This region includes the genes *FYB*, *C9*, and *DAB-2*.

Respiratory function has been associated with *DAB-2*. DAB-2 is a clathrin-associated sorting protein (CLASP) that contributes to clathrin recruitment, vesicle formation, and cargo selection. In the lungs, cystic fibrosis transmembrane conductance regulator, a cAMP-activated chloride channel expressed in the apical plasma membrane of human airway epithelial cells, is endocytosed in a DAB-2-dependent manner [26]. DAB-2 also plays a role in bone morphogenetic protein signaling [27] and nerve cell differentiation [28].

Complement C9 may play a role in skeletal development through its function during endochondral ossification [29], and in respiratory function [30], and has been implicated in demyelination diseases [31]. *FYB* has been linked to osteoclastogenesis [32] and lung physiology [33]. Additionally, a neurologic function has been described for *FYB* through an association with hereditary motor-sensory neuropathy [34].

OSMR plays a key role in bone homeostasis as demonstrated by the OSMR(^-/-^) mouse, which exhibits an osteopetrotic phenotype due to an effect on osteoclast differentiation [35,36]. OSMR has also been implicated in the respiratory system [37], and the nervous system [38], as well as metabolic symptoms such as mature-onset obesity, severe hepatic steatosis, and insulin resistance [39]. Other proteins that play an essential role in the LIFR pathway as ligands or as competing receptor, such as the cytokine receptor-like factor 1 (CRLF1), cardiotrophin-like cytokine factor 1 (CLCF1), and ciliary neurotrophic factor (CNTF) may play a role in STWS [13] and are discussed in more detail below.

## The LIFR protein and its signaling pathway

### *The* LIFR *protein*

**Figure 2:**
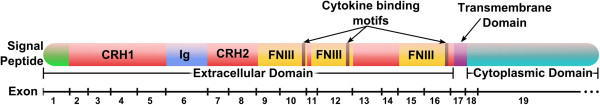
**The LIFR protein with domains and exons shown.** The numbers indicate the location of the 19 exons (figure not to scale). The domains (CRH1, Ig, CRH2, FNIII, TD and CD) are illustrated within their corresponding region of the protein (extracellular, transmembrane, or cytoplasmic). SP: signal peptide; CRH: cytokine receptor homology domain; Ig: Ig-like domain; FNIII: type III fibronectin domain; TM: transmembrane domain; CD: cytoplasmic domain.

The LIFR protein (also called glycoprotein-190; gp 190) is composed of a signal peptide followed by three main domains. The extracellular domain (45-833aa) includes two cytokine receptor homology domains (CRH1 and CRH 2), one Ig-like domain (Ig), and one type III fibronectin domain with three modules (FNIII). Following the extracellular domain is the transmembrane domain (TM; 834-858aa), and finally the cytoplasmic domain (CD; 859-1097aa; Figure 2).

### The LIFR signaling pathway

**Figure 3:**
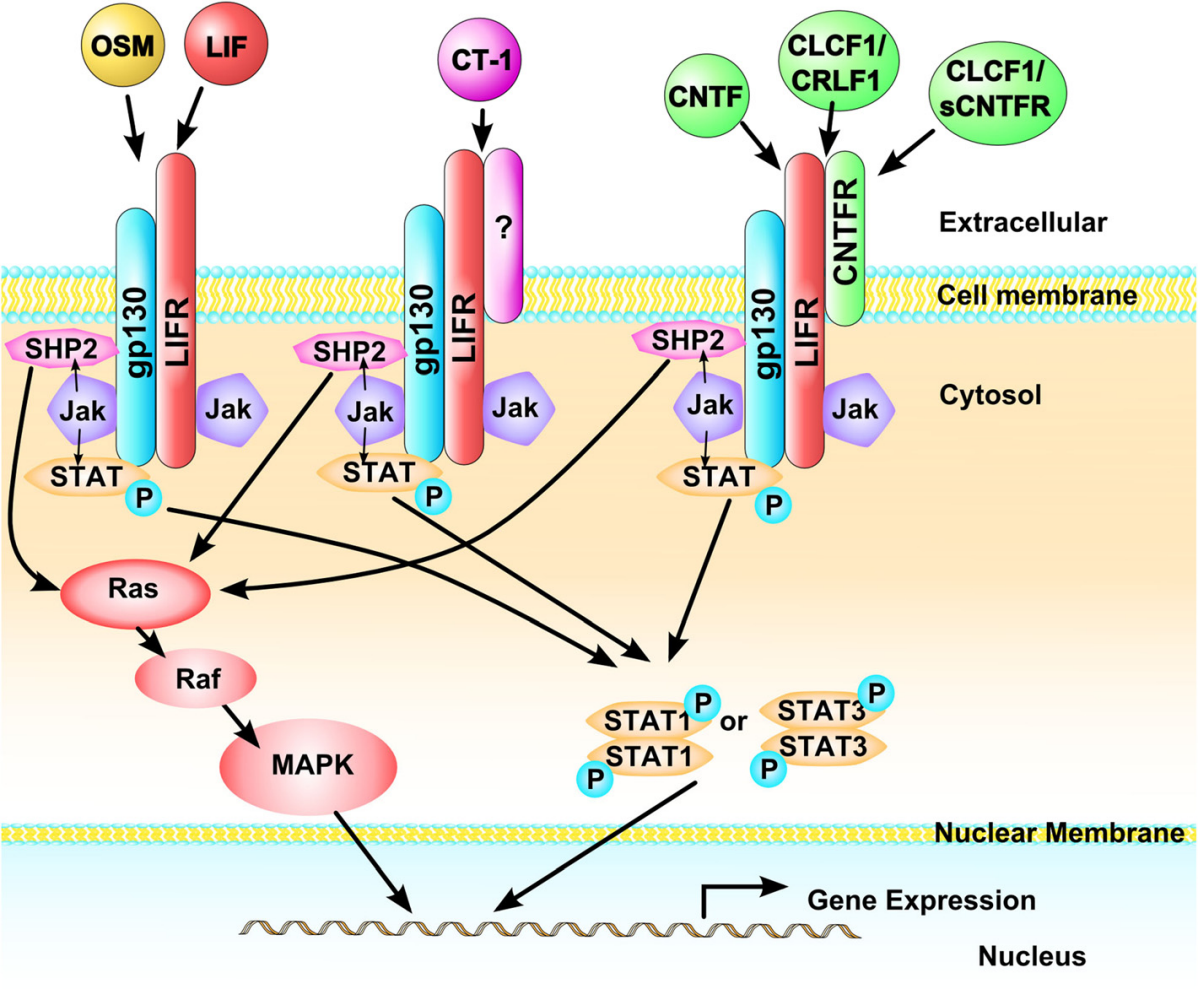
**IL-6 family cytokines that signal through the leukemia inhibitory factor receptor (LIFR).** After cytokine binding, LIFR associates with gp130 to initiate the JAK/STAT1 or JAK/STAT3 pathway. In some cases, the SHP2/RAS/MAPK pathway is initiated. Oncostatin-M (OSM) and leukemia inhibitory factor (LIF) bind to LIFR without any other associated receptors. Cardiotrophin-1 (CT-1) binds to another receptor which associates with LIFR/gp130. Ciliary neurotrophic factor (CNTF) first binds to its receptor (CNTFR), which then recruits LIFR, followed by gp130. Cardiotrophin-like cytokine factor-1 (CLCF-1) binds with either cytokine receptor-like factor-1 (CRLF-1) or soluble ciliary neurotrophic factor receptor (sCNTFR) before binding to CNTFR.

LIFR binds with low affinity to several IL-6 family members, including leukemia inhibitory factor (LIF), oncostatin-M (OSM), cardiotrophin-1 (CT-1, also abbreviated as CTF-1), ciliary neurotrophic factor (CNTF), and cardiotrophin-like cytokine factor 1 (CLCF-1, also abbreviated as CLC) (Figure 3) [40-43]. Both LIF and OSM can bind to the LIFR. LIF binds to LIFRβ, which then recruits gp130 for higher affinity and cell signaling [23]. In contrast, OSM binds gp130 with low affinity but has little to no biological activity unless a second receptor chain is recruited, either the LIFRβ or the more highly specific OSMRβ [44-47]. CT-1 binds to gp130 and LIFR [48], while CNTF first binds to ciliary neurotrophic factor receptor (CNTFRα) before recruiting LIFR. [49] Cardiotrophin-like cytokine factor 1 (CLCF-1) forms a heterodimer with either cytokine receptor-like factor 1 (CRLF1) or soluble ciliary neurotrophic factor receptor (sCNTFR) and competes for this same receptor complex (Figure 3; [13,50-52]).

When a cytokine binds to LIFR (primarily through the fibronectin type III domain), LIFR associates with glycoprotein 130 (gp130), and the addition of gp130 creates a high-affinity complex [53]. The dimerization of LIFR and gp130 causes cytoplasmic JAK to be phosphorylated at the tyrosine 705 residue. JAK then phosphorylates tyrosine residues on the intercellular domains of gp130 and LIFR [54,55]. In turn, STAT3 proteins are phosphorylated [54]. STAT3 then forms a homodimer and moves to the nucleus, where it binds to cytokine-responsive elements and ultimately activates gene transcription (Figure 3) [55-57]. Alternatively, LIFR binding can stimulate JAK/STAT1 in a similar manner [56,57]. To a lesser extent, the SHP2/RAS/MAPK pathway can also be activated (Figure 3) [55,58,59]. LIFR is also present in a soluble form, which binds to LIF in a competitive manner, so that LIF cannot exert its effects on cells [60]. In some cell types, after signaling is completed, LIFR is internalized through endocytosis and degraded [61-63].

## LIFR ligands linked to neonatal development

### Leukemia inhibitory factor receptor ligands: bone remodeling, neuronal development, and myoblast differentiation

LIF is a pleiotropic cytokine, secreted by a variety of cell types, including epithelial and stromal cells of the endometrium [64], osteoblasts [65,66], bone marrow stromal cells, fibroblasts, astrocytes, heart myoblasts, T lymphocytes, monocytes, and thymic epithelial cells among others [67].

The traits of the *Lif* knockout mice that are shared with STWS include giant osteoclasts [68] and some loss of motor neurons [69]. Giant osteoclasts are the result of interactions between FRA2 and c-JUN with LIF [68]. On the other hand, some have argued that there is no severe neuronal dysfunction in *Lif*^*-/-*^ mice [70].

LIF and CT-1 play a role in bone resorption. LIF and CT-1 were found to stimulate the proliferation and differentiation of both osteoblasts and osteoclasts [71-75]. Specifically, LIF and CT-1 stimulate osteoclast formation by enhancing the expression of RANKL (receptor activator of nuclear factor kappa-B ligand). RANKL is the primary mediator of osteoclast formation, function, and survival. Hence, RANKL plays a role in the reduction of bone density, volume and strength. *Ct-1* knockout mice showed osteopenia at the neonatal stage. Taken together, loss of LIF and CT-1 signaling in STWS due to the absence of a functional LIFR most likely contributes to osteopenia and bone-specific traits seen in STWS.

LIF also acts as a neurotrophic factor [76]. The smooth tongue phenotype seen in STWS is due to a lack of fungiform papillae. Fungiform papillae do not develop in the absence of LIF and CNTF signaling that cannot be mediated by the mutated *LIFR* gene [77]. Furthermore, LIF and CNTF are known to stimulate cholinergic differentiation in sympathetic neurons, inducing choline acetyletransferase gene expression, which in turn promotes the survival of cholinergic neurons [78]. Similarly, LIF and CT-1 are important for the cholinergic transdifferentiation of cardiac sympathetic neurons. Hence, it is likely that a lack of LIF and CT-1 downstream signaling leads to the cardiovascular phenotype seen in STWS [79-81]. LIF also has neuromodulary roles in the respiratory airways [82]. Table 2 summarizes the relationship between STWS symptoms and the loss of specific cytokine signaling that cannot be mediated by LIFR due to a mutated *LIFR* gene and absence of LIFR protein.

**Table 2:** Stüve-Wiedemann syndrome (STWS) with the corresponding molecular etiology

**STWS phenotype**	**Cytokine**	**Reference**
Smooth tongue	LIF, CNTF	[7,77]
Osteopenia	LIF, OSM, CT-1	[58,68,89,99]
Cardiovascular malfunctions	LIF, CT-1	[79-81,94,99]
Paradoxical sweating	CLCF1/CRLF1	[9,13,106]
Dysphagia	CT-1?, OSM?, CLCF1/CRLF1?	[20,70,107,108]
Respiratory distress	CT-1?, OSM?	[82]
Short stature	CNTF	[58,70,89,100]

Each of the cytokines signal through the leukemia inhibitory factor receptor (LIFR). In STWS, the *LIFR* gene is mutated, and due to the mutation, cytokine signaling is blocked, and the corresponding manifestations of STWS occur. Only symptoms for which the mechanism has been explored are listed. Those followed by a question mark are still under considerable speculation. LIF: leukemia inhibitory factor; CNTF: ciliary neurotrophic factor; OSM: oncostatin-M; CT-1: cardiotrophin-1; CLCF1: cardiotrophin-like cytokine factor-1; CRLF1: cytokine receptor-like factor-1.

LIF induces skeletal muscle satellite cells to proliferate via the JAK2-STAT3 pathway [83,84]. Satellite cells are quiescent resident multipotential cells that are essential for muscle growth and hypertrophy, and are recruited for muscle growth, regeneration, and repair of injured muscles. LIF is able to both promote or inhibit myoblast differentiation, depending upon conditions [83,85], and acts as a survival factor for myoblasts [86]. Thus, the lack of LIF downstream signaling resulting from an absence of LIFR in STWS may be a contributing factor in the muscular symptoms associated with STWS.

### Oncostatin-M (OSM): bone and motor neuron development

OSM shares similarities with LIF. For example, both are able to induce the differentiation of myeloid leukemia cells to macrophage-like cells in mice [87]. LIF and OSM are located near each other on chromosome 22, and their arrangements suggest that LIF and OSM may be the result of a gene duplication event of an ancestral gene [88].

OSM, CT-1, and to a lesser extent, LIF, are involved in bone formation *in vitro* and *in vivo*[58,89]. OSM can bind to either OSMR:gp130 or LIFR:gp130 heterodimeric receptors. The effect of OSM on bone formation depends on which receptor complex it acts through. When OSM acts through LIFR:gp130 receptor complex , it promotes bone formation by inhibiting sclerostin, for example. Conversely, when OSM acts through OSMR:gp130 receptor complex, it promotes bone resorption by stimulating RANKL production and osteoclast formation [71,90,91]. Therefore, in STWS, the lack of OSM signaling through LIFR:gp130 receptor complex is not able to contribute positively to bone formation, and, in addition, OSM signaling mediated by OSMR:gp130 promotes bone resorption, thus playing a role in osteopenia.

The number of motor neurons in the facial nucleus, lumbar spinal cord, and nucleus ambiguous are severely reduced in mice that do not express LIFR. Importantly for STWS, the nucleus ambiguus innervates the esophagus, pharynx, larynx, and coordinates swallowing. The nucleus ambiguus is responsible for initiating the respiratory rhythm. LIF and CNTF enhance the differentiation and survival of motor neurons, yet Li et al. found that mice deficient in CNTF do not show a decrease in motor neurons, and that mice deficient in LIF do not show any severe neuronal defects. Therefore, Li et al. demonstrated that the reduction in motor neurons seen in STWS is due to the inability of OSM and CT-1 to signal through the mutated LIFR [70]. Taken together, OSM and CT-1 are the most likely LIFR ligands responsible for the respiratory distress and dysphagia seen in STWS. Others have argued that the respiratory distress of STWS may be secondary to myotonia instead of pulmonary hypoplasia [20].

### Cardiotrophin-1 (CT-1): heart, motor neurons, and airway smooth muscle

CT-1, along with LIF, was shown to play an important role in the cholinergic transdifferentiation of cardiac sympathetic neurons in rodents [79,92]. Specifically, CT-1 reduced the number of preganglionic sympathetic neurons, which are important for inducing heart rate, ventricular pressure, and contractility [93]. CT-1 is also known to induce cardiac myocyte hypertrophy and vascular smooth muscle cell proliferation *in vitro*[80]. Therefore, the lack of CT-1 signaling in STWS may play a role in the cardiovascular phenotype [94]. CT-1 was also found to play a role in motor neuron survival [95]. As indicated above, CT-1 plays essential roles in bone and skeletal development. Additionally, a role for CT-1 in airway smooth muscle cells has been identified [96,97]. Therefore, an absence of CT-1 signaling in STWS may contribute to the respiratory phenotype of STWS.

### Ciliary Neurotrophic Factor (CNTF): motor neuron survival and contribution to short stature

CNTF was first isolated from chick eyes, where it helped the survival of embryonic ciliary ganglia [98]. CNTF is expressed in the developing nervous system, particularly in the motor neurons, and it plays a role in motor neuron survival [70].

STWS includes low birth weight and length, delayed growth, and short stature [13,20]. While one symptom of STWS may be dwarfism, since *LIFR*^*-/-*^ mice exhibit dwarfism [89,99], the short stature within the trunk region seen in STWS may, in part, be secondary to progressive scoliosis, however this would not explain the reduction in leg length [13]. Relevant to both explanations, CNTF is expressed in chondrocytes, the cells that are responsible for longitudinal bone growth at the growth plate [89,100]. LIF and CT-1 are also expressed in chondrocytes [101,102], however LIF and CT-1 do not induce chondrogenesis [89]. A short bone phenotype is seen in *Cntf* knockout mice [100]. Therefore, impairment of CNTF signaling in the absence of a functional LIFR in STWS is the most likely cause of the short stature phenotype observed in STWS.

### Cardiotrophin-like Cytokine Factor-1 (CLCF-1): autonomic functioning

Crisponi syndrome and cold-induced sweating syndrome share some features with STWS, such as feeding difficulties, trismus, paradoxical sweating (i.e., sweating with low body temperatures; [9,20]), and hyperthermic episodes [10]. Crisponi syndrome is now considered to be the same disorder as cold-induced sweating syndrome [103], and cold-induced sweating syndrome is caused by mutations in the *CLCF-1* or *CRLF-1* genes [9]. CLCF-1 binds with either CRLF-1 (cytokine receptor-like factor-1) or sCNTFR (soluble ciliary neurotrophic factor receptor) and then competes with CNTF (ciliary neurotrophic factor) for the receptor complex composed of CNTFR, LIFR and gp130 (Figure 3; [51,104,105]). Therefore, it seems that the dysautomonic symptoms seen in STWS are caused by a lack of CLCF-1/CRLF-1 signaling due to a mutated *LIFR* gene [13,21]. Adding to this evidence, in mice, Clcf-1/Crlf-1 appears to be responsible for cholinergic differentiation of neurons innervating sweat glands [106].

Mice lacking Crlf-1, Cntfr and Clcf-1 are unable to suckle and die shortly after birth [107]. These mice also have a reduced number of motor neurons in the facial nucleus [108]. Therefore, a lack of CLCF-1/CRLF-1 signaling is the best candidate for the dysphagia and facial muscle contractions seen in STWS.

## A note about bowing of the long bones

### Myotonia and mechanical forces May play a role

The etiology for each of the major STWS symptoms discussed thus far has been on a cellular level. However mechanical forces also may play an important role in STWS. Congenital bowing of the long bones is a hallmark feature of STWS [1], but it is not unique to STWS and manifests in other syndromes including type IX Ehlers-Danlos syndrome, Campomelic Displasia, Larsen syndrome and other conditions. [109-112]. Some have suggested that prenatal bowing of the long bones results from the mechanical forces of imbalanced muscles acting on structurally weak bones *in utero*[113-115]. Begam et al. found that prior to birth, patients with STWS have myotonia [20], which could produce imbalanced muscular force. Other studies also noted myotonia as a symptom [16,116,117]. Therefore, bowing of the long bones in STWS may be due to mechanical forces acting on bones that are weakened by inadequate signaling.

## Managing Stüve-Wiedemann syndrome

There are no treatments available for STWS at this time. However, aminoglycosides are capable of inducing the read-through of premature stop codons. Since premature stop codons are present in many *LIFR* mutations associated with STWS, the aminoglycoside gentamycin was tested on fibroblast cultures from STWS patients. High doses of gentamycin restored lost JAK/STAT signaling. Although high doses of gentamycin are nephro- and ototoxic *in vivo*, perhaps a similar treatment modality should be explored further [55].

Currently, STWS is managed symptomatically, with prevention of lung aspirations being a top priority [12]. Since STWS patients are unable to maintain a respiratory rhythm or swallow during the first year of life, oftentimes, intubation, nasogastric tube feeding and/or gastrostomy are necessary [118]. Although swallowing abilities generally improve over time [13], it is recommended that caretakers are vigilant while the patient is eating for at least the first five years of life as aspiration and choking can still occur later in childhood [18]. Furthermore, children with STWS have been noted to bite their tongue and cause damage. This can be prevented by using an appliance to cover the teeth to prevent tongue damage until the child gains enough self-awareness to limit unintentional tongue biting [118].

Osteopenia or osteoporosis in STWS may be treated using bisphosphonates with calcium, 1,25 OH vitamin D and/or human growth hormone [18]. Surgery is often necessary to improve other bone malformations [13,119], and physical therapy may also be helpful [118].

In STWS, it is important to protect the eyes from damage, including sunlight, to prevent visual loss. Once ocular issues arise (e.g., corneal opacities, hypolacrimation and impaired pupillary function), a balanced approach is recommended [120]. The results of corneal transplantation in children with anesthetic corneas are poor [121]. However, corneal opacities can be corrected with keratectomy procedures [17]. For hypolacrimation, the frequent use of artificial tear drops and ointment at night are recommended. Additionally, lacrimal punctum dilation and punctual plugs can be implemented to encourage the accumulation of a tear reservoir. Tarsorrhaphies can be performed to help protect the cornea from exposure. Tropicamide eye drops are used to help dilate the pupil. If tropicamide fails, iridectomies are recommended [120].

Prior to surgery, physicians and STWS patients and families should be advised of certain complications which may occur with STWS. Specifically, some have argued that anesthesia increases the risk of malignant hyperthermia in STWS [122,123]. However, others have recently argued against this claim [17]. Additionally, tetraplegia was report in two STWS patients within 40 hours of an otherwise uneventful thoracolumbar scoliosis corrective surgery. Pizones et al. therefore recommended close monitoring after spinal surgery regardless of the course of the surgery. The first symptom of delayed tetraplegia in these cases was acute severe neck pain [4].

## Future directions

### More information about etiology

Although most reported cases of STWS are associated with a mutation in the *LIFR* gene [7], there are some diagnosed cases of STWS in which the patient does not have a *LIFR* mutation [13]. These cases should be explored further. It is possible that those patients were misdiagnosed, as STWS shares many traits with other syndromes [10,20,99]. However, it is also very likely that other genes play a role in STWS [13]. Identical frameshift mutations in the *LIFR* gene in different individuals have also been reported to show differing outcomes or severity [7].

Even in the cases where LIFR signaling is known to be the root cause of STWS, connections between signaling and symptoms have not been fully elucidated. It is known that many cytokines have redundant roles. It is likely to expect that the cytokines interact with one another, and that other proteins involved in STWS indirectly will be discovered with additional research.

### Improving outcomes

It has been noted that STWS patients who survive the first year of life show an improvement in their ability to swallow and regulate breathing [13]. This is a crucial improvement, as feeding difficulties, respiratory failure and hyperthermic episodes are the most frequently cited causes of early death in STWS [6,7,10]. However, it appears that the reason behind this improvement has not yet been explored on a physiological level, or on a molecular level. It would be interesting to learn what causes this shift in STWS phenotypes over time, and what causes these serious symptoms to occasionally return to cause unexpected death. Furthermore, this knowledge could have important implications in the development of treatments which are currently unavailable.

## Conclusions

STWS is a rare bent-bone dysplasia with dysautonomic manifestations that is generally caused by the autosomal recessive inheritance of a mutated *LIFR* gene. The symptoms of STWS are the result of a lack of LIFR signaling, although the exact mechanisms remain unclear for most phenomena. There is currently no treatment available for STWS. Instead, symptoms are managed accordingly. Although STWS is a rare condition, the prognosis remains poor and there are many unanswered questions regarding its pathology. Therefore, further research is needed to provide a better mechanistic understanding as well as to make progress toward novel therapies that take advantage of what we do know about the targeted manipulation of specific signaling pathways.

## Consent

Written informed consent was obtained from the patient, who is now if legal age, for the publication of this report and any accompanying images.

## Abbreviations

CLCF-1 or CLC: Cardiotrophin-like cytokine factor-1; CNTF: Ciliary neurotrophic factor; CNTFR: Ciliary neurotrophic factor receptor; CRLF1: Cytokine receptor-like factor 1; CT-1 or CTF1: Cardiotrophin 1; LIF: Leukemia inhibitory factor; LIFR: Leukemia inhibitory factor receptor; OSM: Oncostatin-M; OSMR: Oncostatin-M receptor; sCNTFR: Soluble ciliary neurotrophic factor receptor; STWS: Stüve-Wiedemann syndrome; SJS2: Schwartz-Jampel syndrome type 2.

## Competing interests

There are no conflicts of interest to declare.

## Authors’ contributions

DM and JTO contributed to the conceptual design. DM carried out data collection. KT designed the review figures. DM created the first draft, which was revised critically by JTO, CJ, and KT. CJ and KT provided analysis and interpretation of information related to IL-6 family members. JTO provided analysis and interpretation related to skeletal development. All authors read and approved the final manuscript.
